# Opportunistic protozoa in HIV seropositive cases and best stool concentration technique for detection

**DOI:** 10.1186/1471-2334-12-S1-P2

**Published:** 2012-05-04

**Authors:** P Sreenivasulu Reddy, P Madhurima, P Vasundhara Devi

**Affiliations:** 1 Department of Microbiology, Narayana Medical College, Nellore, Andhra Pradesh, India

## Introduction

Parasitic gastrointestinal diseases increase morbidity and mortality in HIV patients. This study is aimed at the occurrence of *Cryptosporidium*, *Isospora*, *Cyclospora* and *Microsporidium* in the stool samples of HIV positive cases since the diarrhea is the second most common presentation of HIV positive cases who requires hospitalization.

## Materials and methods

Stool specimen from HIV infected patients (n=100) were included. Each time specimens were divided into two portions of which one was plain and second part mixed with 10% buffered formalin saline in 3:1 ratio. Blood samples were collected for lymphocyte counts. Samples were processed and compared with Formal-Ether sedimentation and Sheather’s sugar floatation technique for the detection of oocysts.

## Results

*Isospora belli* was predominant opportunistic protozoa detected. *Cryptosporidium* oocysts were found in 2 cases of acute diarrhea and one case with chronic diarrhea. No *Cyclospora* and *Microspora* were detected. Sheather’s sugar floatation technique is found better in concentrating the oocysts of Isospora and Cryptosporidium. Along the oocysts, 2 cases of *Ancylostoma duodenale*, one case of each *Giradia lamblia* and *Strongyloides stercoralis* were detected.

## Conclusion

While testing for detection of protozoan parasites from HIV cases, it needs to collect multiple stool samples if feasible. Sheather’s sugar floatation technique is superior to Formal-Ether sedimentation to detect the oocysts of *Cryptosporidium* and *Isospora belli*. Absolute lymphocyte count is probably good when used as a marker for CD4 count assessment where the facilities are not available for CD4 count testing.

